# Ascophyllan Supplementation Is Safe and Associated with Exploratory Modulation of Innate Immune Phenotypes, Biochemical Parameters, and the Gut Microbiome in a Randomized Pilot Trial

**DOI:** 10.3390/md24060213

**Published:** 2026-06-15

**Authors:** Shohei Mizuno, Jorge Luis Espinoza, Lam Quang Vu, Hirona Banno, Yusuke Iida, Saki Shinohara, Do Tung Dac, Yuya Nakagami, Kaori Uchino, Tomohiro Horio, Ichiro Hanamura, Nobuhiro Asai, Megumi Enomoto, Hiroya Tani, Takayuki Nakayama, Susumu Suzuki, Akiyoshi Takami

**Affiliations:** 1 Division of Hematology, Department of Internal Medicine, Aichi Medical University, Nagakute 480-1195, Aichi, Japan; shohei@aichi-med-u.ac.jp (S.M.); quanglamvu1991@gmail.com (L.Q.V.); iida.yuusuke.240@mail.aichi-med-u.ac.jp (Y.I.); yamada.saki.097@mail.aichi-med-u.ac.jp (S.S.); ksakai@aichi-med-u.ac.jp (K.U.); kuroro@aichi-med-u.ac.jp (T.H.); hanamura@aichi-med-u.ac.jp (I.H.); takami-knz@umin.ac.jp (A.T.); 2 Faculty of Health Sciences, Kanazawa University, Kodatsuno 5-11-18, Kanazawa 920-0942, Ishikawa, Japan; dotungdac@stu.kanazawa-u.ac.jp; 3 Department of Central Clinical Laboratory, Aichi Medical University Hospital, Nagakute 480-1195, Aichi, Japan; banno.hirona.880@mail.aichi-med-u.ac.jp (H.B.); nakagami.yuuya.223@mail.aichi-med-u.ac.jp (Y.N.); menomo@aichi-med-u.ac.jp (M.E.); tani.hiroya.116@mail.aichi-med-u.ac.jp (H.T.); 4 Department of Clinical Infectious Diseases, Aichi Medical University, Nagakute 480-1195, Aichi, Japan; asai.nobuhiro.039@mail.aichi-med-u.ac.jp; 5 Department of Blood Transfusion and Clinical Laboratory, Aichi Medical University, Nagakute 480-1195, Aichi, Japan; tnaka@aichi-med-u.ac.jp; 6 Research Creation Support Center, Aichi Medical University, Nagakute 480-1195, Aichi, Japan; suzukis@aichi-med-u.ac.jp

**Keywords:** ascophyllan, natural killer cells, innate immunity, gut microbiome, functional dietary polysaccharide

## Abstract

Background: Ascophyllan, a sulfated polysaccharide extracted from brown seaweed, has shown immunomodulatory and antioxidant effects in preclinical studies, yet human clinical evidence remains scarce. This randomized, double-blind, placebo-controlled pilot trial evaluated the safety and exploratory biological effects of daily ascophyllan supplementation in healthy adults. Methods: Twelve participants were randomized to receive either ascophyllan (*n* = 6) or placebo (*n* = 6) for 28 days. Safety was monitored through adverse event reporting and repeated laboratory assessments, including hematology, biochemistry, and inflammatory markers. Immune cell populations were analyzed via serial flow cytometry, serum total antioxidant capacity was measured at multiple time points, and gut microbiome composition was profiled using 16S rRNA gene sequencing. All analyses were exploratory in nature. Results: Ascophyllan supplementation proved well tolerated, with no adverse events observed and stable hematologic, renal, and biochemical parameters throughout the study. Exploratory longitudinal analyses suggested directional modulation of NK-cell-associated phenotypes during ascophyllan supplementation, including directional changes in CD57^+^, NKp46^+^, and NKG2D^+^ NK-cell phenotypes; however, group × time interaction analyses did not remain statistically significant after correction for multiple comparisons. Serum antioxidant capacity showed inter-individual variability with a directional but non-significant increase in the ascophyllan group at intermediate time points. Exploratory microbiome analyses suggested modest directional compositional differences involving members of the *Bacteroidaceae* and *Bifidobacteriaceae* families; however, no taxon remained statistically significant after correction for multiple comparisons. Conclusions: These preliminary findings indicate that ascophyllan is safe and well tolerated in healthy adults and may be associated with modulation of innate immune phenotypes, subtle microbiome compositional differences, and directional changes in antioxidant capacity. Larger, adequately powered clinical trials are warranted to confirm these observations and further investigate potential biological and clinical effects.

## 1. Introduction

Marine-derived polysaccharides have attracted increasing interest as functional dietary components due to their diverse biological activities, including immunomodulatory, antioxidant, and anti-inflammatory properties. Among these, sulfated polysaccharides from brown seaweeds (Phaeophyceae) stand out for their ability to modulate innate immune responses, influence redox balance, and modulate host–microbe interactions [1,2]. Ascophyllan is a fucose-containing sulfated heteropolysaccharide extracted from the brown seaweed *Ascophyllum nodosum* (commonly known as knotted wrack or egg wrack), a species abundant in the intertidal zones of the North Atlantic Ocean. Structurally related to but distinct from fucoidans, ascophyllan features a unique monosaccharide composition (including fucose, xylose, glucuronic acid, and others), a notable sulfate content, and a relatively lower molecular weight (typically in the range of 100–300 kDa for native forms). These characteristics contribute to its specific bioactivities, which often differ in potency or mechanism from those of classical fucoidans [3,4].

Preclinical studies have highlighted ascophyllan’s promising immunostimulatory profile. It potently activates macrophages via induced secretion of tumor necrosis factor-α (TNF-α) and granulocyte colony-stimulating factor (G-CSF) [5]. It also promotes natural killer (NK)-cell cytotoxicity in several models [6], and enhances T-cell immune functions via dendritic cell maturation and cytokine secretion [7,8]. We recently demonstrated that ascophyllan directly activates human NK cells in vitro via mTOR pathway activation, resulting in enhanced cytotoxic activity against Epstein–Barr virus (EBV)–infected B cells [9]. These findings provide mechanistic evidence that ascophyllan can potentiate NK cell–mediated immunity and support its translational relevance in humans.

In parallel with direct immune modulation, accumulating evidence indicates that dietary polysaccharides may exert indirect immunological effects through the gut microbiome [10]. Specific microbial taxa, including members of *Bacteroidaceae* and *Bifidobacteriaceae*, have been associated with enhanced innate immune responses and NK cell function [11,12,13], highlighting the potential for microbiome-mediated immune regulation.

With growing interest in safe, food-derived approaches to support immune resilience, particularly in aging populations and in the context of environmental stressors and post-infectious immune dysfunction [14], ascophyllan emerges as a promising nutraceutical candidate. However, translating robust preclinical findings into human applications remains essential, as animal and in vitro models cannot fully recapitulate the complexity of human immune physiology, dietary interactions, or long-term tolerability. Accordingly, comprehensive clinical evaluation is needed to confirm safety, define dose-dependent effects on immune phenotypes, and explore potential interactions between direct immunostimulatory activity and gut microbiome modulation.

Despite encouraging preclinical data, human clinical evidence regarding the safety and immunological effects of ascophyllan remains limited. In particular, no randomized controlled trials have comprehensively evaluated its impact on innate immune cell subsets, systemic antioxidant capacity, and gut microbiome composition in healthy individuals. Therefore, the present randomized, placebo-controlled pilot study was designed to evaluate its short-term safety profile and explore its potential effects on immune phenotypes, biochemical and hematologic parameters, antioxidant capacity, and gut microbiome composition. By integrating immunophenotyping with exploratory microbiome analysis, this study aimed to generate foundational clinical data to inform the design of future mechanistic and efficacy trials of ascophyllan as a functional immunomodulatory dietary component.

## 2. Results

### 2.1. Study Population and Compliance

A total of twelve healthy adult volunteers were enrolled and randomized to receive either ascophyllan (*n* = 6) or placebo (*n* = 6). All participants completed the study protocol, and adherence to supplementation was confirmed throughout the intervention period. Baseline demographic characteristics, including age, sex distribution, and body weight, were comparable between the two groups (Table 1).

### 2.2. Safety and Tolerability

Ascophyllan supplementation was well tolerated over the 28-day intervention period. No adverse events, side effects, or clinically relevant symptoms were reported by participants in either the ascophyllan or placebo groups. No participants discontinued the study due to tolerability concerns. The absence of reported adverse effects supports the short-term safety of ascophyllan administration in healthy adults.

### 2.3. Laboratory Safety and Hematologic Stability

Across the 28-day intervention period and follow-up to Day 43, complete blood count (CBC) indices and biochemical parameters remained stable in both treatment groups, with no evidence of clinically meaningful hematologic (Supplementary Table S2A) or biochemical toxicity (Supplementary Table S2B).

Formal longitudinal comparisons between the ascophyllan and placebo groups were performed using linear mixed-effects models with participant-specific random intercepts to account for repeated measurements over time. For key hematologic safety endpoints, including white blood cell (WBC) count and platelet (PLT) count, no statistically significant group-by-time interactions were observed, indicating comparable hematologic trajectories between treatment arms (Table 2).

Group-level trajectories for WBC, hemoglobin, and platelets showed only modest within-range fluctuations over time and did not indicate treatment-related leukocytosis or cytopenias (Supplementary Table S3A). Similarly, biochemical markers of hepatic function (AST, ALT), renal function (creatinine), and systemic inflammation (CRP) demonstrated no statistically significant differences in longitudinal patterns between groups in mixed-effects analyses (Supplementary Table S3B). A transient increase in AST was observed at Day 8 in the ascophyllan group (18.50 ± 3.51 U/L at baseline versus 28.00 ± 22.93 U/L at Day 8). Nevertheless, AST concentrations remained within the normal reference range, were not accompanied by elevations in ALT, and were not sustained at later study visits, including Day 29 (19.50 ± 4.09 U/L) and Day 43 (18.67 ± 2.25 U/L) (Supplementary Table S3B). The clinical significance of this isolated finding is therefore uncertain. Consistent with this observation, AST did not demonstrate a significant group-by-time interaction in longitudinal modeling, supporting the absence of a clinically meaningful hepatic effect. Other routine chemistry measures, including albumin, LDH, lipid profiles, glucose, and CRP, remained stable over time and comparable between groups, with only minor expected variability (Supplementary Table S3B). No laboratory abnormalities necessitated discontinuation of supplementation, and no adverse events were reported.

Collectively, these findings indicate that ascophyllan supplementation was not associated with statistically or clinically significant alterations in key hematologic or biochemical safety parameters, supporting its favorable short-term safety and tolerability in healthy adults.

### 2.4. Effects of Ascophyllan on Immune Cell Populations

Flow cytometric analyses were performed to evaluate the effects of ascophyllan supplementation on circulating lymphocytes and innate immune cell subsets over time.

#### 2.4.1. Natural Killer Cells

**Figure 1:**
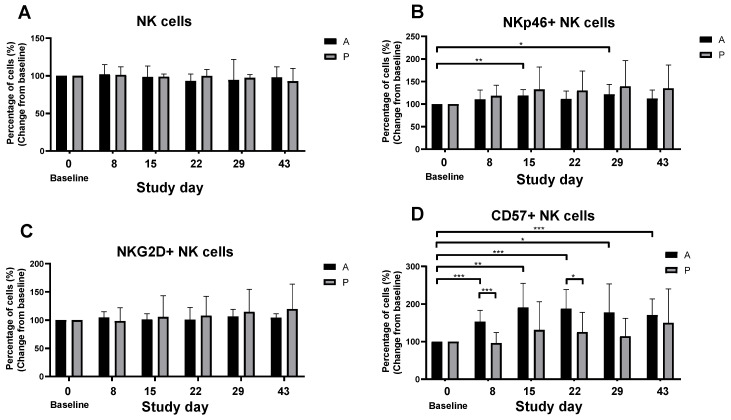
Exploratory longitudinal modulation of NK-cell-associated phenotypes during ascophyllan supplementation. Longitudinal flow cytometric analysis of circulating NK-cell subsets in healthy adults randomized to ascophyllan (A; *n* = 6) or placebo (P; *n* = 6) for 28 days. (**A**) Frequency of total NK cells. (**B**) Frequency of NKp46^+^ NK cells. (**C**) Frequency of NKG2D^+^ NK cells. (**D**) Frequency of CD57^+^ NK cells. Data are shown over time at Days 0, 8, 15, 22, 29, and 43 and are presented as mean ± standard deviation (SD). Exploratory longitudinal analyses suggested directional changes in CD57^+^ and NKp46^+^ NK-cell phenotypes during ascophyllan supplementation, whereas NKG2D expression remained relatively stable over time. *p* values shown in the figure reflect exploratory within-group comparisons relative to baseline. (*** indicates *p* = 0.001; ** indicates *p* = 0.01; * indicates *p* = 0.05).

Exploratory longitudinal analyses suggested directional modulation of NK-cell-associated phenotypes during ascophyllan supplementation. Ascophyllan supplementation was associated with directional increases in CD57^+^ and NKp46^+^ NK-cell frequencies at selected time points relative to baseline, whereas NKG2D expression remained relatively stable over time (Figure 1).

Mixed-effects group × time interaction analyses demonstrated nominal longitudinal differences for selected NK-cell markers; however, none remained statistically significant after correction for multiple comparisons. Specifically, NKG2D demonstrated a nominal interaction signal (*p* = 0.047; FDR q = 0.140), whereas CD57 and NKp46 did not demonstrate statistically significant group × time interactions. These findings should therefore be interpreted as exploratory immunophenotypic observations rather than definitive evidence of enhanced NK-cell function (Table 3 and Supplementary Figure S3).

#### 2.4.2. Dendritic Cell Subsets

**Figure 2:**
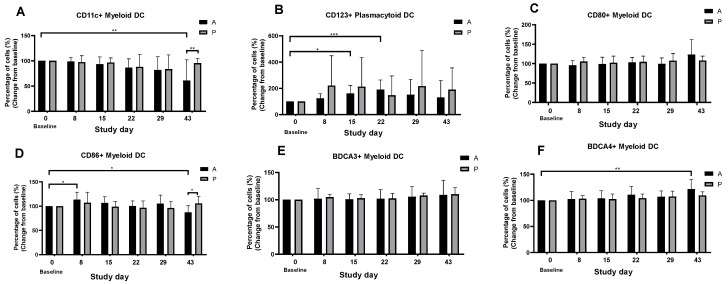
Exploratory changes in dendritic cell subsets during ascophyllan supplementation. Longitudinal analysis of circulating dendritic cell subsets. (**A**) Frequency of CD11c^+^ myeloid dendritic cells (mDCs). (**B**) Frequency of CD123^+^ plasmacytoid dendritic cells (pDCs). (**C**) Frequency of CD80^+^ myeloid dendritic cells. (**D**) Frequency of CD86^+^ myeloid dendritic cells. (**E**) Frequency of BDCA3^+^ myeloid dendritic cells. (**F**) Frequency of BDCA4^+^ myeloid dendritic cells. Ascophyllan supplementation was associated with a transient increase in pDCs, peaking at intermediate time points, whereas total mDCs remained largely stable. A modest late increase in BDCA4^+^ mDCs was observed at Day 43 in the ascophyllan group. Data are expressed as percentage of parent populations (mean ± SD). (*** indicates *p* = 0.001; ** indicates *p* = 0.01; *indicates *p* = 0.05).

Among dendritic cell populations, exploratory within-group analyses suggested a transient increase in plasmacytoid dendritic cells (CD123^+^ pDCs), peaking at Day 22 before returning toward baseline levels. Myeloid dendritic cells (CD11c^+^ mDCs) remained largely stable over time, although a modest late increase in BDCA4^+^ mDCs was observed at Day 43 in the ascophyllan group (Figure 2). Formal mixed-effects group × time interaction analyses did not identify statistically significant longitudinal differences between the ascophyllan and placebo groups for either pDCs or myeloid DCs (Supplementary Table S4). These findings should therefore be interpreted as descriptive and hypothesis-generating rather than evidence of a definitive treatment-related dendritic cell effect (Figure 2).

#### 2.4.3. T and B Lymphocytes

**Figure 3:**
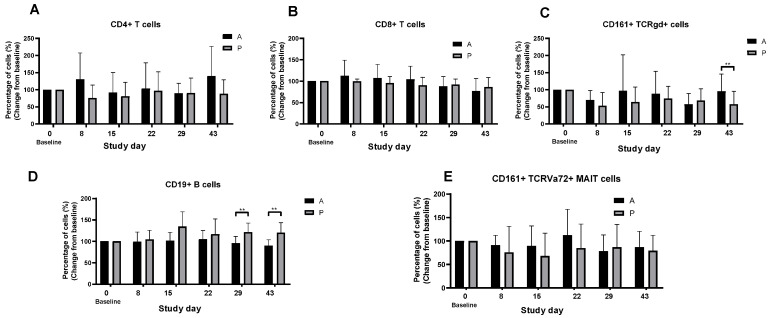
Adaptive lymphocyte populations remain stable during ascophyllan supplementation. Longitudinal frequencies of adaptive immune cell subsets in ascophyllan- and placebo-treated participants. (**A**) CD4^+^ T cells. (**B**) CD8^+^ T cells. (**C**) CD161^+^ γδ T cells. (**D**) CD19^+^ B cells. (**E**) MAIT cells. No consistent or statistically significant changes were observed in either group over the study period. Data are presented as mean ± SD across study visits (Days 0–43). (** indicates *p* = 0.01).

No significant changes were observed in the frequencies of CD4^+^ or CD8^+^ T cells, CD19^+^ B cells, MAIT cells, or γδ T cells in either treatment group during the study period (Figure 3).

Collectively, these findings indicate that ascophyllan supplementation may be associated with modulation of selected innate immune phenotypes, particularly NK cell maturation and plasmacytoid dendritic cell abundance, without inducing broad alterations in adaptive lymphocyte populations.

### 2.5. Effects on Serum Antioxidant Capacity

**Figure 4:**
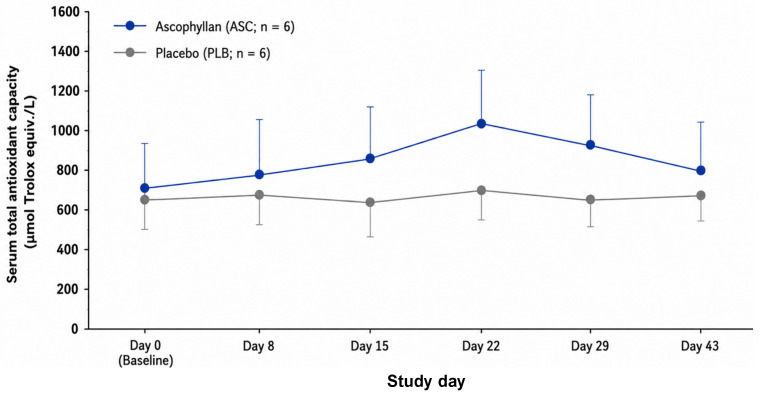
Serum total antioxidant capacity during ascophyllan supplementation. Longitudinal serum total antioxidant capacity was measured at baseline and follow-up study visits in participants receiving ascophyllan (ASC; *n* = 6) or placebo (PLB; *n* = 6). Data are presented as mean ± standard deviation (SD). Participants receiving ascophyllan demonstrated a directional increase in antioxidant capacity relative to baseline, most apparent at Day 22, whereas changes in the placebo group were smaller and less consistent over time.

Serum total antioxidant capacity was measured longitudinally at all scheduled study visits. Considerable inter-individual variability was observed in both groups. Participants receiving ascophyllan demonstrated a directional increase in antioxidant capacity relative to baseline, most apparent at Day 22, whereas changes in the placebo group were smaller and less consistent over time. Although some participants receiving ascophyllan demonstrated directional increases in antioxidant capacity at intermediate time points, these findings should be interpreted cautiously given the pilot nature of the study (Figure 4).

Nevertheless, exploratory mixed-effects analyses of serum total antioxidant capacity values did not identify statistically significant group × time interactions between the ascophyllan and placebo groups (Group × Time β = 0.318, Standard Error = 0.287, *p* = 0.279). However, given the pilot nature of the study, these findings should be interpreted cautiously.

### 2.6. Effects of Ascophyllan Supplementation on the Gut Microbiome

**Figure 5:**
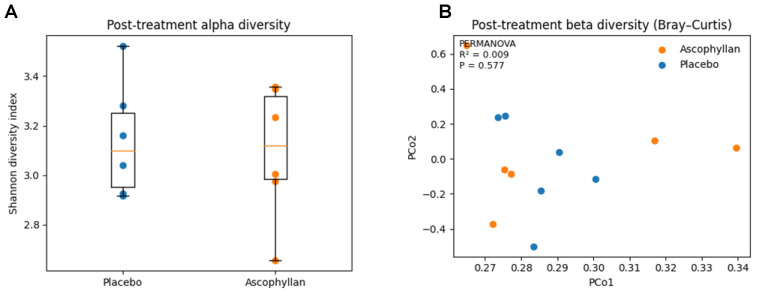
Exploratory gut microbiome analyses following ascophyllan supplementation. (**A**) Alpha diversity measured by the Shannon index at post-treatment time points, comparing ascophyllan and placebo groups. No statistically significant difference in within-sample diversity was observed. (**B**) Principal coordinates analysis (PCoA) of Bray–Curtis dissimilarity demonstrating overlap in overall microbial community structure between treatment groups. PERMANOVA analysis did not detect significant differences in global composition (R^2^ = 0.009, *p* = 0.577). Together, these analyses indicate that short-term ascophyllan supplementation was not associated with major alterations in global microbiome diversity but may be accompanied by subtle compositional shifts at the taxon level (see Supplementary Figure S4 and Supplementary Table S5).

Exploratory analyses were performed to assess the effects of ascophyllan supplementation on gut microbiome composition using post-treatment samples only. Overall microbial alpha diversity, assessed by the Shannon index, did not differ significantly between the ascophyllan and placebo groups, indicating comparable within-sample diversity following the intervention (Figure 5A). Beta diversity analysis based on Bray–Curtis dissimilarity demonstrated substantial overlap in overall community structure between the two groups on principal coordinates analysis (PCoA). Consistent with this visual pattern, permutational multivariate analysis of variance (PERMANOVA) did not identify a significant difference in global microbial composition between ascophyllan- and placebo-treated participants (R^2^ = 0.009, *p* = 0.577; Figure 5B).

Despite the absence of marked differences in global diversity metrics, taxon-specific exploratory analyses suggested modest compositional shifts associated with ascophyllan supplementation. At higher taxonomic levels, ascophyllan treatment was directionally associated with relative enrichment of *Bacteroidetes* and *Actinobacteria* and a relative reduction in Firmicutes compared with placebo. These trends were reflected at lower taxonomic levels, with increases observed in Bacteroidia- and Actinobacteria-associated lineages and relative decreases in several Firmicutes-related groups. At the genus level, differential abundance analysis identified several taxa showing directional relative differences in the ascophyllan group, including *Parvimonas*, *Butyricicoccus*, and *Roseburia*, whereas genera such as *Klebsiella* and *Akkermansia* were relatively more abundant in the placebo group (Supplementary Figure S4). However, effect sizes were modest, and no individual taxon remained statistically significant after correction for multiple comparisons.

Collectively, these findings indicate that short-term ascophyllan supplementation was not associated with major alterations in overall gut microbiome diversity or structure but may be accompanied by subtle, taxon-specific compositional shifts that warrant confirmation in larger, adequately powered studies (Supplementary Table S5).

### 2.7. Integrated Summary of Findings

In this randomized pilot study, ascophyllan supplementation was safe and well tolerated in healthy adults. Exploratory analyses suggested directional modulation of selected innate immune phenotypes and subtle microbiome compositional differences; however, these findings did not remain statistically significant after correction for multiple comparisons. Collectively, these preliminary observations support the feasibility of ascophyllan supplementation and provide a foundation for future larger mechanistic studies.

## 3. Discussion

In this randomized pilot clinical trial, ascophyllan supplementation was safe and well tolerated in healthy adults and associated with exploratory modulation of innate immune cell populations and accompanied by coherent, biologically plausible changes in gut microbiome composition. Although exploratory in nature, these findings provide the first integrated human evidence linking ascophyllan intake with innate immune activation and microbiome remodeling.

A key observation of this study was the directional modulation of NK-cell phenotypes, particularly increases in CD57^+^ and NKp46^+^ NK cells following ascophyllan supplementation. Selective increases in CD57^+^ and NKp46^+^ NK cells are consistent with a shift toward a more mature and functionally competent NK-cell phenotype, as CD57 is widely used as a marker of terminal NK-cell maturation [15,16], while NKp46 is a key activating receptor involved in antiviral and antitumor responses [17,18,19]. Although exploratory longitudinal analyses suggested directional increases in these phenotypes in participants receiving ascophyllan, formal mixed-effects group × time interaction analyses did not remain statistically significant after correction for multiple comparisons. Importantly, the absence of statistical significance should be interpreted cautiously given the pilot nature of the study and the very limited sample size, which substantially restricted statistical power to detect modest longitudinal immune effects. Therefore, these findings should be interpreted as preliminary immunophenotypic observations rather than definitive evidence of enhanced NK-cell function. Nevertheless, the directional changes observed are biologically plausible and consistent with prior preclinical studies of sulfated polysaccharides, which have been reported to enhance innate immune readiness without inducing excessive inflammatory activation.

In parallel, ascophyllan supplementation was associated with a transient expansion of plasmacytoid dendritic cells (pDCs), peaking at intermediate time points before returning toward baseline. pDCs play a critical role in bridging innate and adaptive immunity through type I interferon production and antiviral surveillance [20,21]. The temporal nature of this response suggests a regulated immunological effect rather than sustained immune stimulation, supporting the safety profile observed in this study. Importantly, no consistent changes were detected in adaptive immune compartments, including T cells, B cells, MAIT cells, or γδ T cells, further indicating that ascophyllan’s immunological impact is selective and primarily innate.

Exploratory gut microbiome analyses suggested modest, directionally consistent compositional shifts associated with ascophyllan supplementation. Although overall alpha diversity and global community structure did not differ significantly between treatment groups, taxon-specific analyses indicated relative enrichment of *Bacteroidetes*- and *Actinobacteria*-associated lineages in the ascophyllan group, accompanied by relative reductions in selected Firmicutes-related taxa compared with placebo. At lower taxonomic resolution, several genera exhibited differential relative abundance favoring the ascophyllan group, whereas others were more abundant in placebo-treated participants. These patterns were subtle, did not persist after correction for multiple comparisons, and should therefore be interpreted as hypothesis-generating observations rather than definitive treatment effects. Nevertheless, these microbiome changes are biologically plausible and align with the immune phenotype observed [22]. The gut microbiota plays a fundamental role in the development, education, and maintenance of the human immune system, with commensal microorganisms shaping both innate and adaptive immune responses through microbial metabolites, antigenic stimulation, and modulation of mucosal barrier integrity. Specific bacterial taxa have been implicated in the regulation of dendritic cell maturation, NK-cell activity, and cytokine production, thereby influencing antiviral defense and systemic immune balance. Conversely, dysbiosis has been associated with impaired immune regulation, chronic inflammation, and increased susceptibility to infectious diseases [23,24]. Members of *Bifidobacteriaceae* and *Bacteroidaceae* modulate innate immunity by enhancing NK cell cytotoxicity, regulating DC maturation, and producing SCFAs/tryptophan derivatives that shape immune signaling [12,25]. Conversely, specific *Firmicutes*-associated taxa have been linked to immune tolerance or dampened cytotoxic responses in certain contexts [26,27]. While none of the observed microbiome changes remained statistically significant after correction for multiple comparisons, the consistency of directionality across taxonomic levels strengthens the biological relevance of these findings.

The biological plausibility of these exploratory findings may also relate to the unique structural characteristics of ascophyllan as a marine-derived sulfated heteropolysaccharide. Unlike classical fucoidans, ascophyllan contains a heterogeneous composition of fucose-rich glycans, xylose, uronic acids, and sulfate groups, which may contribute to distinct immunological and physicochemical properties [28]. Previous studies have suggested that sulfation degree, monosaccharide composition, molecular conformation, and uronic acid content critically influence the capacity of marine polysaccharides to interact with immune receptors and modulate downstream signaling pathways [29]. Sulfated fucose-containing polysaccharides have been reported to regulate dendritic cell maturation, macrophage activation, NK-cell activity, and cytokine production through pathways involving pattern-recognition receptors and intracellular signaling cascades, including NF-κB and mTOR-associated mechanisms [20,21,30,31]. Although the present study was not designed to evaluate structure–function relationships directly, the observed exploratory immunophenotypic and microbiome-associated findings are consistent with previously described biological activities of sulfated marine polysaccharides.

The antioxidant capacity analyses revealed considerable inter-individual variability, as expected in a small pilot cohort. Notably, participants receiving ascophyllan demonstrated a directional increase in serum antioxidant capacity relative to baseline, most apparent at the intermediate time point (Day 22), whereas changes in the placebo group were smaller and less consistent. Although between-group differences did not reach statistical significance, this temporal pattern suggests a potential systemic antioxidant effect associated with ascophyllan supplementation. Given the known antioxidant properties of marine-derived polysaccharides [32,33], future studies incorporating complete longitudinal sampling and oxidative stress biomarkers will be essential to clarify this aspect of ascophyllan’s biological activity [34].

From a safety perspective, longitudinal monitoring of complete blood count and biochemical parameters provides additional reassurance regarding the tolerability of ascophyllan supplementation. Hematologic indices, including total white blood cell counts and differential cell populations, remained stable over time, with no evidence of treatment-related leukocytosis, cytopenias, or inflammatory activation. Similarly, renal function markers and most biochemical parameters showed no clinically meaningful changes throughout the intervention and follow-up periods. A transient numerical increase in aspartate aminotransferase was observed at an early time point in the ascophyllan group; however, values remained within the normal reference range, were not accompanied by parallel elevations in alanine aminotransferase, and resolved spontaneously at subsequent visits. In the absence of sustained enzyme elevation, concurrent inflammatory changes, or clinical symptoms, this isolated fluctuation is unlikely to reflect hepatocellular injury and is of uncertain biological significance. However, future studies incorporating larger cohorts and more comprehensive hepatic monitoring will be important to further characterize potential hepatic effects. Importantly, no participants reported adverse events, and no laboratory abnormalities necessitated discontinuation of supplementation. Collectively, these findings support the short-term safety of ascophyllan in healthy adults and indicate that the observed immunological and microbiome-associated effects occurred without detectable hematologic or biochemical toxicity.

Several limitations should be acknowledged. First, the small sample size and pilot design limited statistical power and precluded definitive hypothesis testing. Second, all analyses should be considered exploratory and hypothesis-generating. Third, no direct functional assays of NK-cell activity or cytokine production were performed, limiting mechanistic interpretation of the observed immunophenotypic changes. Fourth, although microbiome analyses identified directional compositional differences, no individual taxon remained statistically significant after correction for multiple comparisons. Fifth, although compositional characterization was performed prior to study initiation, detailed structural analyses such as molecular conformation assessment and sulfation-pattern mapping were beyond the scope of the present pilot study. Sixth, only a single oral dose of ascophyllan was evaluated, precluding dose–response assessment, and the study population consisted exclusively of healthy adults, potentially limiting generalizability to individuals with underlying immune or metabolic disorders. Seventh, although baseline fecal samples were collected, microbiome analyses focused on post-treatment comparisons and therefore do not permit assessment of within-subject temporal changes. Future studies incorporating longitudinal microbiome modeling will be important to better define treatment-related microbial dynamics.

**Figure 6:**
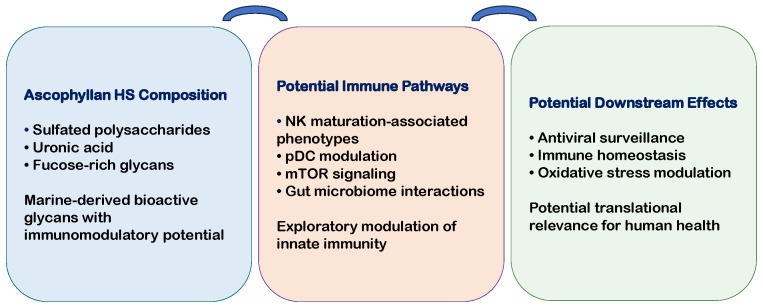
Proposed mechanisms linking ascophyllan supplementation, innate immune modulation, antioxidant activity, and gut microbiome interactions.

Despite these limitations, this study has notable strengths. It represents the first randomized human trial to integrate safety, immunophenotyping, antioxidant assessment, and microbiome analysis in the context of ascophyllan supplementation. The convergence of innate immune modulation and microbiome shifts provides a compelling mechanistic framework for future investigations. Collectively, these exploratory findings support a conceptual framework in which ascophyllan-derived sulfated polysaccharides may interact with innate immune pathways, redox biology, and gut microbiome composition in an interconnected manner (Figure 6).

Conceptual schematic summarizing exploratory biological pathways potentially associated with ascophyllan supplementation in healthy adults. Ascophyllan HS contains sulfated polysaccharides, uronic acid, and fucose-rich glycans that may influence innate immune signaling and host–microbiome interactions. Proposed intermediary pathways include modulation of NK-cell maturation-associated phenotypes, transient plasmacytoid dendritic cell (pDC) expansion, mTOR-associated immune signaling, and microbiome-related immunomodulatory interactions. These mechanisms may collectively contribute to downstream biological effects including enhanced antiviral surveillance, maintenance of immune homeostasis, and modulation of oxidative stress responses. Given the pilot nature of the study, these proposed mechanisms should be considered exploratory and hypothesis-generating rather than definitive causal pathways.

In conclusion, ascophyllan supplementation was safe and well tolerated in healthy adults and was associated with exploratory modulation of innate immune phenotypes, including directional changes in NK-cell maturation-associated markers and a transient expansion of plasmacytoid dendritic cells. However, these immune findings did not remain statistically significant after correction for multiple comparisons and should therefore be interpreted cautiously, given the pilot nature and limited sample size of the study, which substantially restricted statistical power to detect modest longitudinal immune effects. Exploratory analyses further suggested subtle, biologically plausible microbiome compositional differences involving taxa previously linked to immunomodulatory functions, although no individual taxon remained statistically significant after multiple-testing correction. In addition, ascophyllan supplementation was associated with directional but non-significant changes in systemic antioxidant capacity at intermediate time points, accompanied by considerable inter-individual variability. Collectively, these findings support the short-term feasibility and tolerability of ascophyllan supplementation and provide a rationale for larger, adequately powered clinical studies incorporating comprehensive immune functional assays, oxidative stress biomarkers, dose-escalation strategies, and high-resolution microbiome profiling to further elucidate its mechanisms of action and potential clinical utility.

## 4. Materials and Methods

### 4.1. Study Design and Participants

This phase 1, randomized, double-blind, placebo-controlled trial (jRCT: jRCTs041230038) enrolled 12 healthy Japanese volunteers (6 males and 6 females; median age 34 years [range 29–54]; median body weight 56 kg [range 45–73]). The study was approved by the Institutional Review Board of Aichi Medical University Hospital (approval number: 2022-CR002) and was conducted in accordance with the Declaration of Helsinki. All participants provided written informed consent prior to enrollment. Participants were randomized in a 1:1 ratio to receive either ascophyllan (*n* = 6) or placebo (*n* = 6). Randomization was performed prior to study initiation, and both participants and investigators were blinded to treatment allocation. All 12 participants completed the study protocol. Inclusion criteria were: age 20–60 years, absence of subjective symptoms, normal liver and renal function tests, and alcohol consumption fewer than four times per week. Exclusion criteria included long-term use of any medication, presence of chronic disease, history of invasive cancer within the past 5 years, history of non-invasive cancer within the past year, and smoking within the past year.

Healthy adults were selected for this pilot study to establish preliminary human safety and feasibility data prior to future studies in populations with immune or metabolic disorders. Participants were instructed to maintain their usual dietary habits throughout the study period and to avoid initiation of new nutritional supplements or major dietary changes during the intervention.

Ascophyllan HS powder, extracted from *Ascophyllum nodosum*, was encapsulated for oral administration and was prepared by Hayashikane Sangyo Co., Ltd. (Shimonoseki, Yamaguchi, Japan). Each active capsule contained 100 mg of Ascophyllan HS, 71.3 mg of crystalline cellulose (excipient), and 13.7 mg of anti-caking agents (10.0 mg calcium stearate and 3.7 mg fine-particle silicon dioxide). Placebo capsules contained 171.3 mg of starch and 13.7 mg of the same anti-caking agents.

Compositional characterization of Ascophyllan HS was performed by the manufacturer prior to study initiation using standardized analytical procedures. Neutral monosaccharide composition was determined following acid hydrolysis and ABEE derivatization using HPLC. Monosaccharide standards were used for peak identification and quantification. Representative chromatograms are shown in Supplementary Figure S1. Detailed chromatographic conditions are provided in the Supplementary Methods. Uronic acid content was quantified using the carbazole–sulfuric acid method, whereas sulfate content was determined using a turbidimetric assay. Compositional values are shown in Supplementary Table S1.

Participants were instructed to take one capsule daily after lunch for 28 consecutive days. Compliance and adverse events were evaluated weekly. Adverse events were graded according to the National Cancer Institute Common Terminology Criteria for Adverse Events (CTCAE), version 5.0 [35]. The primary objective of the study was to evaluate the safety of ascophyllan in healthy Japanese adults. The secondary objective was to assess its potential effects on circulating lymphocytes and various metabolic and serological parameters.

### 4.2. Intervention

**Figure 7:**
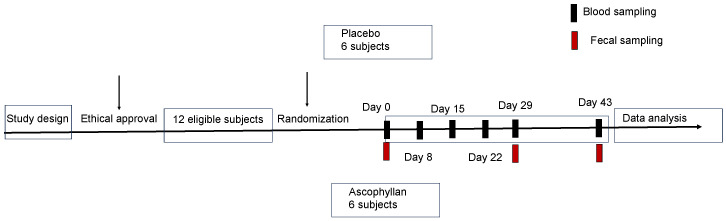
The outline of the study design and sample collection for analysis.

Participants assigned to the intervention group received ascophyllan supplementation daily for 28 days, while the control group received a matched placebo over the same period. The dose and formulation of ascophyllan were selected conservatively for this first-in-human pilot study based on prior preclinical immunomodulatory observations, feasibility considerations, and safety-oriented dose selection. The 28-day duration was selected to evaluate short-term tolerability and exploratory biological responses while minimizing participant burden. Compliance with supplementation was monitored throughout the study period. An overview of the study design is shown in Figure 7.

### 4.3. Study Schedule and Sample Collection

Peripheral blood samples were collected at baseline (Day 0) and at prespecified follow-up time points (Days 8, 15, 22, 29, and 43) for immunological and biochemical analyses. Stool samples were collected for gut microbiome assessment at baseline and post-intervention time points according to the study protocol (Days 0, 29, and 43).

### 4.4. Safety and Tolerability Assessment

Safety and tolerability were assessed throughout the study via participant self-report and investigator inquiry at each study visit. Participants were specifically queried regarding the occurrence of adverse events, gastrointestinal symptoms, or other subjective side effects. Structured laboratory assessments, including complete blood count and routine biochemical testing, were performed longitudinally throughout the study period. All adverse events reported were documented and evaluated for potential relationship to study supplementation.

### 4.5. Flow Cytometric Analysis of Immune Cell Subsets

Peripheral blood mononuclear cells were analyzed using multiparameter flow cytometry to assess lymphocyte and innate immune cell populations. The following immune subsets were evaluated:

NK cells and NK cell phenotypes, including CD57^+^, NKp46^+^, and NKG2D^+^ NK cells. Dendritic cell subsets, including plasmacytoid dendritic cells (CD123^+^ pDCs) and myeloid dendritic cells (CD11c^+^ mDCs). T lymphocyte subsets (CD4^+^, CD8^+^), B cells (CD19^+^), mucosal-associated invariant T (MAIT) cells, and γδ T cells. Cells were stained with fluorochrome-conjugated monoclonal antibodies according to standard protocols. Data were acquired using a calibrated flow cytometer and analyzed using dedicated flow cytometry software. Immune cell populations were expressed as percentages of parent populations. Further details on gating strategies and flow cytometry analysis are shown in Supplementary Figure S2.

### 4.6. Measurement of Serum Antioxidant Capacity

Serum antioxidant capacity was measured using a commercially available total antioxidant capacity assay (Antioxidant Assay Kit, Lot number: 709001, Cayman Chemical, 1180 E. Ellsworth Road—Ann Arbor, MI 48108, USA), performed according to the manufacturer’s instructions. Serum samples were assayed in duplicate, and antioxidant capacity values were calculated using standard curves generated during each assay run. Serum antioxidant capacity measurements were available for all participants at all scheduled time points. Longitudinal comparisons between groups were subsequently evaluated using exploratory mixed-effects modeling with participant-specific random intercepts.

### 4.7. Statistical Analysis

All analyses were conducted as exploratory analyses, given the pilot nature of the study and limited sample size. The primary independent variable was treatment assignment (ascophyllan vs. placebo), while dependent variables included longitudinal immune cell subset frequencies, hematologic and biochemical laboratory parameters, antioxidant capacity measurements, and microbiome diversity metrics. Continuous variables were summarized using means ± standard deviations or medians and ranges as appropriate. Longitudinal analyses were performed using linear mixed-effects models with participant-specific random intercepts to account for repeated measurements over time. Group × time interaction terms were used to evaluate differential longitudinal trajectories between treatment groups. False discovery rate (FDR) correction was applied for selected multiple-comparison analyses. Due to limited statistical power, nominal *p* values and directional trends were interpreted cautiously and considered hypothesis-generating.

Alpha diversity was assessed using the Shannon diversity index calculated from species-level (level-7) relative abundance data. Group differences were evaluated using non-parametric Wilcoxon rank-sum tests. Beta diversity was evaluated using Bray–Curtis dissimilarity, and community-level differences were visualized by principal coordinates analysis (PCoA). Group differences in overall microbial community composition were assessed using permutational multivariate analysis of variance (PERMANOVA) with 999 permutations. Taxon-specific analyses were performed using analysis of variance (ANOVA)–based approaches applied to summarized relative abundance data to identify taxa exhibiting differential abundance between ascophyllan and placebo groups at post-treatment. Summary statistics included test statistics, raw *p* values, and *p* values adjusted for multiple comparisons using false discovery rate (FDR) and Bonferroni correction methods. Given the limited sample size, effect sizes and directional trends were emphasized, and statistical significance was interpreted cautiously. For microbiome analyses, results were interpreted primarily in terms of global diversity patterns and taxon-specific directional changes rather than definitive inferential conclusions. Although stool samples were collected at baseline, the microbiome analysis presented here focused on post-treatment comparisons because of the pilot nature of the study, limited sample size, and the exploratory objective of identifying treatment-associated compositional differences. Baseline-adjusted longitudinal microbiome analyses were not prespecified and will be considered in future adequately powered studies.

A two-sided *p* value < 0.05 was considered nominally significant for exploratory purposes. All statistical analyses and data visualizations were performed using standard statistical software.

## Figures and Tables

**Table 1:** Baseline demographic characteristics of study participants.

ID	Treatment	Sex	Age	Weight (Kg)
22	Ascophyllan	male	29	68
24	Ascophyllan	male	35	68.8
26	Ascophyllan	male	40	56.8
31	Ascophyllan	female	45	52.2
32	Ascophyllan	female	30	48.3
35	Ascophyllan	female	25	48.6
21	Placebo	male	26	73.1
23	Placebo	male	33	71.6
25	Placebo	male	41	63.2
33	Placebo	female	23	45.5
34	Placebo	female	54	45.5
36	Placebo	female	44	55.2

**Table 2:** Exploratory mixed-effects longitudinal analyses of hematologic safety parameters.

Endpoint	Group × Time	Group × Time (FDR)	Δ Ascophyllan (Day 29)	Δ Placebo (Day 29)	* Difference-in-Differences
WBC	0.1478	0.3589	1.2167	−0.55	1.7667
PLT	0.2414	0.4936	17.0	−7.5	24.5

Abbreviations: PLTs: platelets, WBCs: white blood cells. Footnotes: Δ indicates change from baseline. *p*-values for longitudinal group-by-time interactions were derived from likelihood ratio tests in linear mixed-effects models with random intercepts for participants. False discovery rate (FDR) correction was applied across endpoints. * Difference-in-differences (DiD) represents the difference in mean change from baseline between the ascophyllan and placebo groups. Thus, positive DiD values indicate greater increases (or smaller decreases) in the ascophyllan group compared with placebo.

**Table 3:** Exploratory longitudinal mixed-effects analyses of NK-cell-associated immune phenotypes.

Immune Phenotype	Group × Time β	*p* Value	FDR q Value
CD57^+^ NK cells	−0.245	0.715	0.715
NKp46^+^ NK cells	0.516	0.105	0.158
NKG2D^+^ NK cells	0.442	0.047	0.140

Footnote: Values represent exploratory group × time interaction coefficients derived from linear mixed-effects models with participant-specific random intercepts. False discovery rate (FDR) correction was applied across immune phenotypes. No immune phenotype remained statistically significant after correction for multiple comparisons.

## Data Availability

The data that support the findings of this study are available from the corresponding author upon reasonable request.

## Supplementary Materials

The following supporting information can be downloaded at: https://www.mdpi.com/article/10.3390/md24060213/s1, Supplementary Methods; Figures S1: Representative HPLC chromatograms of monosaccharide standards and Ascophyllan HS hydrolysates; Figure S2: The gating strategy for analyzing distinct cellular subsets, including NK cells, B cells, T cells, plasmacytoid dendritic cells, MAIT cells, and gamma delta T cells; Figure S3: Exploratory longitudinal mixed-effects analyses of immune phenotypes; Figure S4: Taxonomic differences in gut microbiota composition between ascophyllan- and placebo-treated participants; Table S1: Chemical composition of Ascophyllan HS preparation; Table S2A: Longitudinal complete blood count parameters in Ascophyllan group; Table S2B: Longitudinal complete blood count parameters in control group; Table S3A: Longitudinal Biochemical parameters in Ascophyllan group; Table S3B: Longitudinal Biochemical parameters in control group; Table S4: Exploratory longitudinal mixed-effects analyses of dendritic-cell subsets; Table S5: Summary of exploratory microbiome compositional analyses.

## Abbreviations

The following abbreviations are used in this manuscript:

ALT	Alanine aminotransferase
ANOVA	Analysis of variance
AST	Aspartate aminotransferase
CBC	Complete blood count
CRP	C-reactive protein
EBV	Epstein–Barr virus
FDR	False discovery rate
LDH	Lactate dehydrogenase
MAIT	Mucosal-associated invariant T
mDC	Myeloid dendritic cell
mTOR	Mechanistic target of rapamycin
NK	Natural killer
PCoA	Principal coordinates analysis
pDC	Plasmacytoid dendritic cell
PERMANOVA	Permutational multivariate analysis of variance
PLT	Platelet
SCFA	Short-chain fatty acid
WBC	White blood cell
