# Supplementary material for: Ascophyllan Supplementation Is Safe and Associated with Exploratory Modulation of Innate Immune Phenotypes, Biochemical Parameters, and the Gut Microbiome in a Randomized Pilot Trial
Source: Mar Drugs. 2026 Jun 15;24(6):213. doi: 10.3390/md24060213 (PMC13301280; doi:10.3390/md24060213)
Supplement: Supplementary file 1 [file marinedrugs-24-00213-s001.zip › marinedrugs-4285750-supplementary.pdf]

**Supplementary materials for “Ascophyllan Supplementation Is Safe and Associated with Exploratory Modulation of Innate Immune Phenotypes, Biochemical Parameters, and the Gut Microbiome in a Randomized Pilot Trial.”**

# Supplementary Methods

### Gut Microbiome Analysis

Gut microbiome composition in fecal samples was assessed using 16S rRNA gene sequencing. Sample processing and sequencing were performed by Takara Bio Inc. (Shiga, Japan). In brief, genomic DNA was extracted from fecal samples using the MoBio PowerLyzer PowerSoil DNA Isolation Kit (MoBio Laboratories, Carlsbad, CA, USA) according to the manufacturer’s instructions. The V4 hypervariable region of the bacterial 16S rRNA gene was amplified using modified universal bacterial primers as follows:

**515F** (5’-TCGTCGGCAGCGTCAGATGTGTATAAGAGACAGGTGCCAGCMGCCGCGGTAA-3’)

**806R** (5’-GTCTCGTGGGCTCGGAGATGTGTATAAGAGACAGGGACTACHVGGGTWTCTAAT-3’), which included Illumina adapter overhang sequences.

Amplicons were generated, purified, indexed, and sequenced in accordance with the Illumina MiSeq 16S Metagenomic Sequencing Library Preparation protocol, with minor modifications. Sequencing reads were demultiplexed and assigned to individual samples based on barcode sequences using the open-source MOTHUR software package. Low-quality reads, sequencing errors, and chimeric sequences were removed through standard denoising and quality-control procedures. High-quality sequences were clustered into operational taxonomic units (OTUs) at 97% sequence identity using the CD-HIT-OTU pipeline. Taxonomic assignment of OTUs was performed using the Ribosomal Database Project (RDP) Classifier. Microbial community composition was summarized at multiple taxonomic levels, including phylum, class, order, family, genus, and OTU.

## Supplementary Figure S1

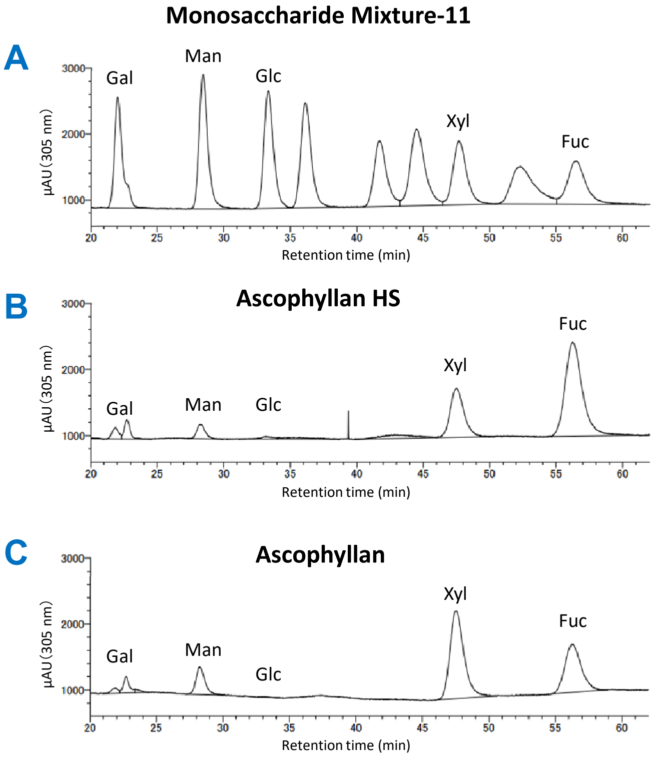


**Analysis conditions**

Nexera™ HPLC system(Autosampler: SIL-40C , Detector:SPD-M40); Shimadzu Corporation, Kyoto, Japan

Column: InertSustain C18(3μm, 4.6mm, 150mm) ;GL Sciences Inc., Tokyo, Japan

Sample labeling method: ABEE Labeling Kit ;MITSUBISHI GAS CHEMICAL COMPANY, INC ., Tokyo, Japan

Solvent: A; 0.2M potassium borate buffer (pH 8.9) / acetonitrile (93/7)
B; 0.02% TFA / acetonitrile (50/50)

Program: 0→100 min (B concentration 0%):Analysis
100→105 min (B concentration 100%):Washing
105→125 min (B concentration 0%):Equilibration
Flow rate: 1.0 mL/min
Temperature: 30℃
Detection: UV (absorbance at 305nm)
Injection volume: 10 μL

### Representative HPLC chromatograms of monosaccharide standards and Ascophyllan HS hydrolysates.

(A) Representative chromatographic separation of ABEE-derivatized monosaccharide standards used for compositional analysis, including galactose (Gal), mannose (Man), glucose (Glc), xylose (Xyl), and fucose (Fuc).

(B) Representative HPLC chromatogram of acid-hydrolyzed Ascophyllan HS demonstrating corresponding monosaccharide peaks identified by retention-time matching with analytical standards.

(C) Representative chromatogram of ascophyllan reference material analyzed under identical chromatographic conditions.

Analyses were performed using a Nexera™ HPLC system (Shimadzu Corporation, Kyoto, Japan) equipped with an InertSustain C18 column following ABEE derivatization. Detection was performed by ultraviolet absorbance at 305 nm. Neutral sugar composition was analyzed following acid hydrolysis, whereas uronic acid and sulfate contents were quantified separately using carbazole and turbidimetric assays, respectively. These analyses were performed as manufacturer-provided quality-control characterization prior to study initiation.

## Supplementary Figure S2. Gating strategies for flow cytometry analysis and data acquisition

### NK subset

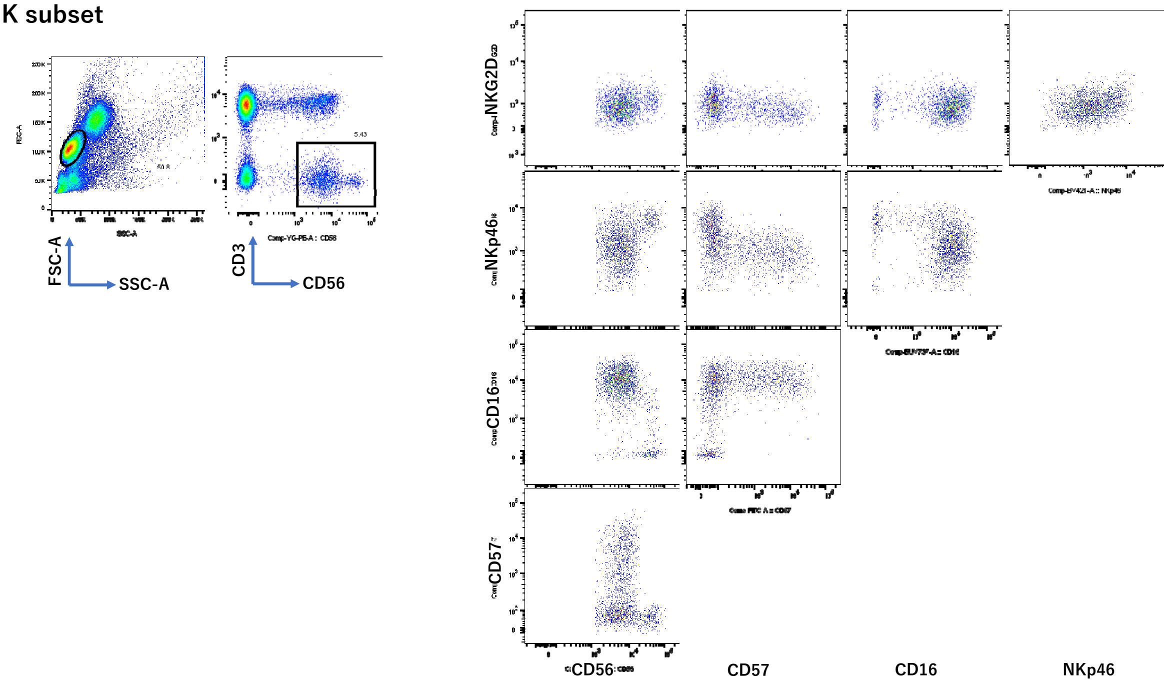


### DC subset

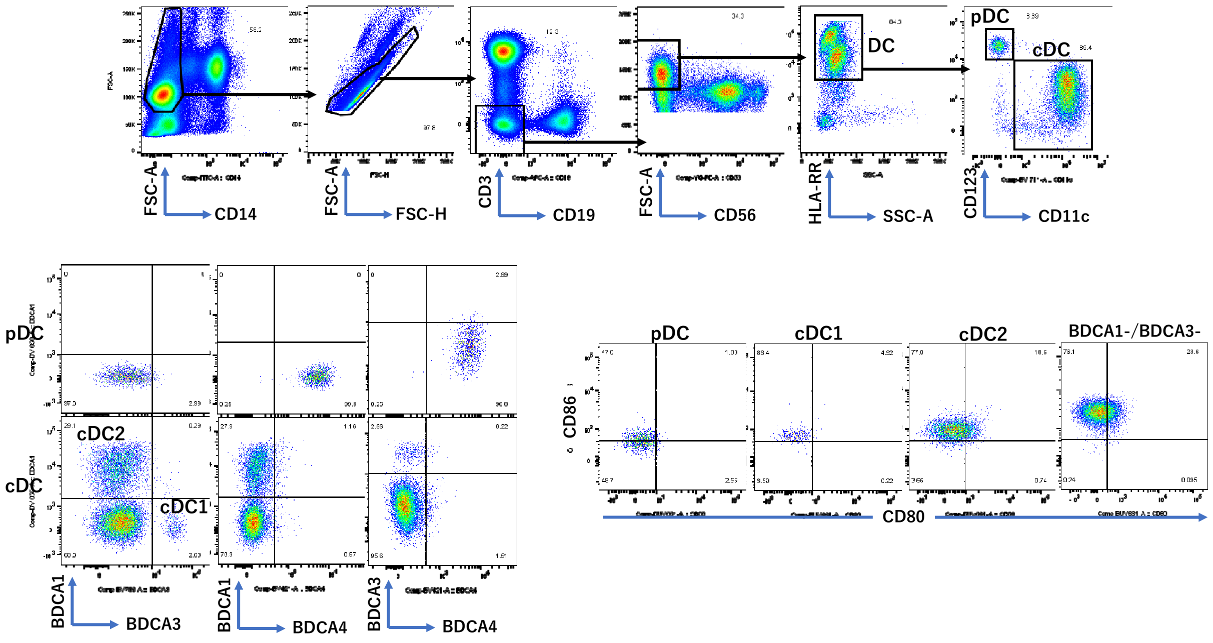

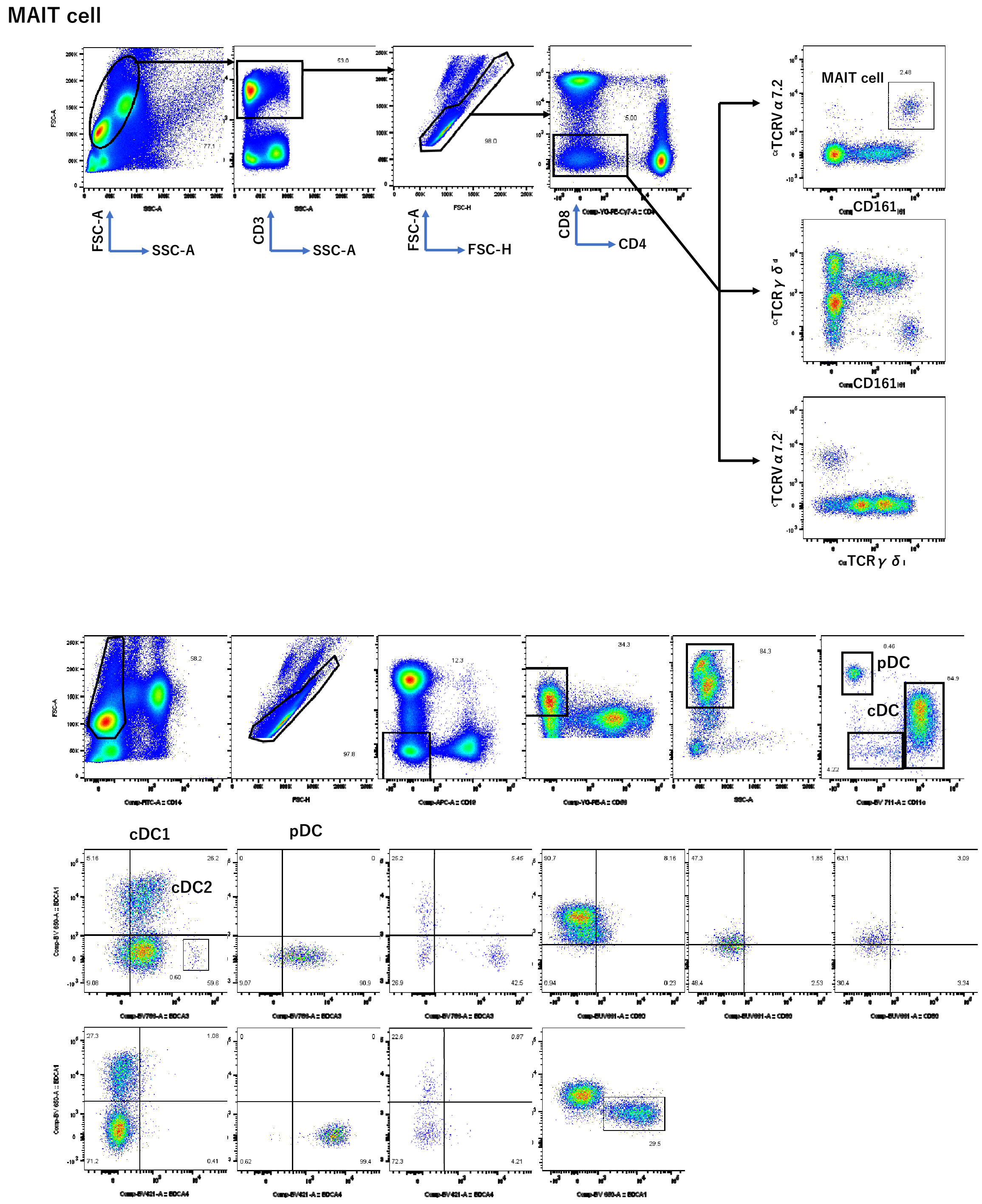


**Supplementary Figure S2.** The gating strategy for analyzing distinct cellular subsets, including NK cells, B cells, T cells, plasmacytoid dendritic cells, MAIT cells, and gamma delta T cells.

## Supplementary Figure S3.

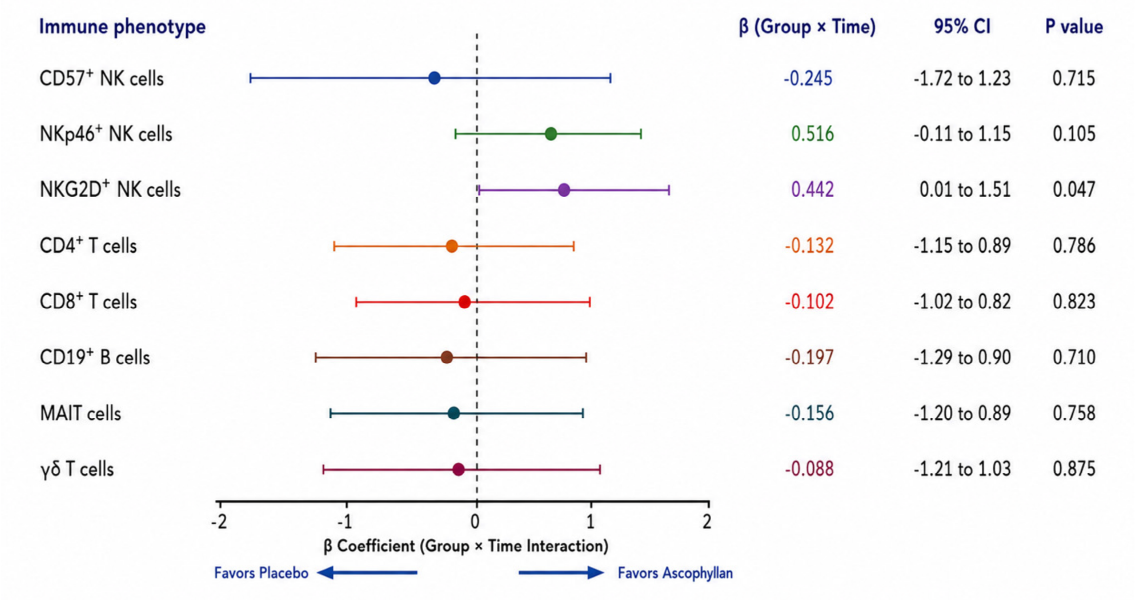


**Supplementary Figure S3. Exploratory longitudinal mixed-effects analyses of immune phenotypes.**

Forest plot summarizing exploratory linear mixed-effects analyses evaluating group×time interactions for longitudinal immune-cell phenotypes in participants receiving ascophyllan or placebo. Points represent β coefficients derived from mixed-effects models with participant-specific random intercepts, and horizontal lines indicate 95% confidence intervals (CI). Positive β values indicate directional longitudinal changes favoring the ascophyllan group, whereas negative values indicate directional changes favoring placebo. Corresponding nominal P values are shown for each immune phenotype. Immune subsets analyzed included $CD57^+$ NK cells, $NKp46^+$ NK cells, $NKG2D^+$ NK cells, $CD4^+$ T cells, $CD8^+$ T cells, $CD19^+$ B cells, mucosal-associated invariant T (MAIT) cells, and γδ T cells. Although $NKG2D^+$ NK cells demonstrated a nominal interaction signal, no immune phenotype remained statistically significant after correction for multiple comparisons (false discovery rate $q > 0.05$). These analyses should therefore be interpreted as exploratory and hypothesis-generating given the pilot nature and limited sample size of the study.

## Supplementary Figure S4.

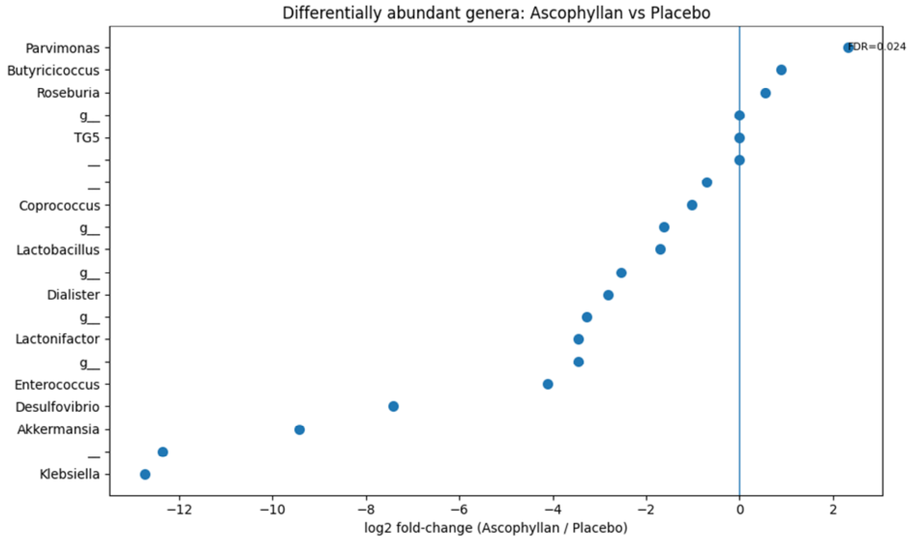


**Supplementary Figure S4.** Taxonomic differences in gut microbiota composition between ascophyllan- and placebo-treated participants.
Differential abundance analysis was conducted at the genus level using post-treatment samples. $Log_2$ fold changes represent relative enrichment in the ascophyllan group compared with placebo. Taxa are ranked by false discovery rate (FDR)–adjusted P values derived from ANOVA, and the top differentially abundant genera are shown. Positive values indicate higher relative abundance in the ascophyllan group, whereas negative values indicate enrichment in the placebo group.

## Supplementary Table S1. Chemical composition of Ascophyllan HS preparation

| Component | Content (%) |
|---|---|
| Fucose | 13.1 |
| Xylose | 4.6 |
| Glucose | 0.2 |
| Mannose | 0.8 |
| Galactose | 0.9 |
| Uronic acid | 21.7 |
| Sulfate | 12.3 |

**Footnote:** Neutral sugar composition was analyzed by high-performance liquid chromatography (HPLC) following acid hydrolysis and ABEE derivatization. Monosaccharide peaks were identified by retention-time matching with analytical standards. Uronic acid content was measured using the carbazole–sulfuric acid assay, whereas sulfate content was determined using a turbidimetric method. Values represent manufacturer-provided quality-control compositional characterization performed prior to study initiation. Significant digits were standardized to reflect analytical precision and avoid overinterpretation of nominal variability. Representative chromatograms are shown in Supplementary Figure S1.

## Supplementary Table S2A. Longitudinal complete blood count parameters in Ascophyllan group

| Time point | WBC | RBC | Hb | PLT | Neu | Ly | Mo |
|---|---|---|---|---|---|---|---|
| Day 0 | 5.47 (0.73) | 4.47 (0.44) | 13.47 (0.90) | 232.33 (26.78) | 55.33 (5.91) | 33.80 (2.89) | 5.57 (1.53) |
| Day 8 | 6.00 (1.88) | 4.55 (0.35) | 13.65 (0.85) | 234.33 (33.16) | 58.98 (8.02) | 30.82 (5.89) | 5.62 (2.31) |
| Day 15 | 6.05 (1.31) | 4.46 (0.52) | 13.50 (1.32) | 252.67 (67.04) | 63.33 (11.19) | 28.37 (7.73) | 4.45 (1.49) |
| Day 22 | 5.23 (0.85) | 4.37 (0.45) | 13.27 (1.15) | 255.33 (69.37) | 54.98 (8.78) | 35.00 (6.62) | 4.85 (1.42) |
| Day 29 | 6.68 (2.18) | 4.38 (0.38) | 13.25 (0.91) | 249.33 (52.43) | 59.25 (11.78) | 30.70 (9.23) | 5.87 (1.28) |
| Day 43 | 6.08 (0.51) | 4.30 (0.58) | 12.92 (1.37) | 244.83 (35.16) | 59.42 (4.71) | 31.58 (2.96) | 5.25 (1.56) |

**Supplementary Table S2B. Longitudinal complete blood count parameters in control group**

| Time point | WBC | RBC | Hb | PLT | Neu | Ly | Mo |
|---|---|---|---|---|---|---|---|
| Day 0 | 6.10 (1.71) | 4.56 (0.48) | 14.20 (1.06) | 215.00 (52.08) | 61.05 (7.67) | 30.62 (7.40) | 5.62 (1.87) |
| Day 8 | 5.53 (2.00) | 4.44 (0.54) | 13.63 (1.26) | 216.33 (59.97) | 56.63 (8.81) | 33.48 (7.80) | 6.07 (2.58) |
| Day 15 | 6.08 (2.61) | 4.50 (0.44) | 13.93 (1.31) | 208.33 (54.46) | 57.10 (6.67) | 33.45 (5.23) | 5.45 (1.91) |
| Day 22 | 6.07 (1.67) | 4.37 (0.48) | 13.60 (1.32) | 204.50 (51.00) | 57.48 (6.66) | 32.42 (6.63) | 6.33 (2.96) |
| Day 29 | 5.55 (2.04) | 4.31 (0.50) | 13.23 (1.28) | 207.50 (54.87) | 56.63 (9.77) | 33.05 (8.63) | 6.37 (3.60) |
| Day 43 | 6.28 (1.48) | 4.30 (0.50) | 13.25 (1.21) | 206.50 (46.52) | 62.30 (12.13) | 28.60 (11.04) | 5.62 (2.05) |

**Abbreviations**: WBC: white blood cells count, RBC: red blood cells count, Hb: hemoglobin, PLT: platelets count, Neu: neutrophils count, Ly: lymphocytes count, Mo: monocytes count

**Supplementary Table S3A. Longitudinal Biochemical parameters in Ascophyllan group**

| Day | AST | ALT | CRE | ALB | CRP | GLU | TG | LDL-C | HDL-C | LD |
|---|---|---|---|---|---|---|---|---|---|---|
| Day 0 | 18.50 (3.51) | 15.67 (7.20) | 0.68 (0.23) | 4.72 (0.21) | 0.05 (0.03) | 92.67 (5.61) | 64.50 (28.26) | 109.50 (32.12) | 71.50 (13.10) | 155.00 (15.47) |
| Day 8 | 28.00 (22.93) | 22.17 (15.98) | 0.69 (0.20) | 4.67 (0.38) | 0.62 (1.19) | 96.83 (7.83) | 78.83 (60.41) | 106.50 (24.17) | 68.17 (13.56) | 162.00 (18.15) |
| Day 15 | 44.00 (5.84) | 25.00 (17.93) | 0.69 (0.21) | 4.83 (0.49) | 0.07 (0.04) | 101.83 (20.89) | 75.83 (44.84) | 121.67 (30.09) | 67.50 (9.52) | 184.83 (56.92) |
| Day 22 | 19.50 (5.01) | 17.17 (4.83) | 0.68 (0.20) | 4.68 (0.16) | 0.04 (0.02) | 92.83 (3.13) | 74.33 (38.07) | 116.33 (24.74) | 71.50 (10.48) | 165.50 (9.81) |
| Day 29 | 19.50 (4.09) | 16.33 (5.82) | 0.67 (0.21) | 4.70 (0.33) | 0.06 (0.07) | 91.00 (5.18) | 68.33 (26.55) | 113.50 (23.20) | 72.00 (13.08) | 164.00 (21.65) |
| Day 43 | 18.67 (2.25) | 16.00 (2.37) | 0.69 (0.24) | 4.60 (0.43) | 0.04 (0.04) | 89.33 (8.04) | 56.17 (28.04) | 108.17 (28.44) | 67.33 (8.98) | 162.67 (18.33) |

**Supplementary Table S3B. Longitudinal Biochemical parameters in control group**

| Day | AST | ALT | CRE | ALB | CRP | GLU | TG | LDL-C | HDL-C | LD |
|---|---|---|---|---|---|---|---|---|---|---|
| Day 0 | 20.17 (3.31) | 18.17 (5.98) | 0.67 (0.22) | 4.50 (0.28) | 0.06 (0.04) | 94.67 (2.34) | 96.67 (62.98) | 105.00 (21.36) | 64.83 (11.89) | 163.83 (34.47) |
| Day 8 | 20.33 (4.46) | 18.83 (5.78) | 0.65 (0.21) | 4.47 (0.35) | 0.03 (0.01) | 88.83 (6.49) | 68.00 (26.57) | 102.83 (24.88) | 66.17 (11.65) | 162.17 (33.82) |
| Day 15 | 20.50 (3.99) | 18.67 (8.09) | 0.65 (0.21) | 4.65 (0.27) | 0.05 (0.04) | 89.50 (2.43) | 68.67 (27.47) | 106.83 (27.80) | 70.67 (8.45) | 167.17 (43.10) |
| Day 22 | 21.00 (4.98) | 18.83 (6.79) | 0.64 (0.24) | 4.53 (0.29) | 0.08 (0.09) | 94.50 (14.32) | 58.83 (17.13) | 101.00 (22.07) | 69.50 (11.27) | 170.33 (36.05) |
| Day 29 | 19.83 (5.19) | 17.50 (5.54) | 0.66 (0.24) | 4.47 (0.24) | 0.05 (0.03) | 93.50 (5.96) | 53.00 (25.83) | 100.00 (19.81) | 69.33 (10.11) | 157.50 (37.81) |
| Day 43 | 19.50 (6.44) | 16.00 (8.15) | 0.66 (0.24) | 4.53 (0.36) | 0.23 (0.44) | 91.50 (7.31) | 68.33 (20.79) | 100.33 (22.68) | 71.00 (9.14) | 163.33 (47.20) |

**Abbreviations**: AST: Aspartate aminotransferase; ALT; Alanine aminotransferase; CRE: creatinine; ALB: Albumin; CRP: C-reactive protein; GLU: Glucose; TG: Triglyceride; LDL-C: Low-Density Lipoprotein Cholesterol; HDL-C: High-Density Lipoprotein Cholesterol; LD: Lactate Dehydrogenase.

## Supplementary Table S4. Exploratory longitudinal mixed-effects analyses of dendritic-cell subsets

| Dendritic-cell phenotype | Group × Time β | 95% CI | P value | FDR q value |
|---|---|---|---|---|
| Plasmacytoid DC | −0.003 | −0.043 to 0.037 | 0.881 | 0.881 |
| Myeloid DC | −0.044 | −0.149 to 0.060 | 0.407 | 0.814 |

**Footnote:** Values represent exploratory group × time interaction coefficients derived from linear mixed-effects models with participant-specific random intercepts. Study visit was modeled as an ordinal longitudinal variable. False discovery rate correction was applied across dendritic-cell phenotypes. No dendritic-cell subset demonstrated a statistically significant group × time interaction.

## Supplementary Table S5. Summary of exploratory microbiome compositional analyses.

| Taxonomic level | Taxon | Direction | Δ Ascophyllan | Δ Placebo | DiD | Nominal P |
|---|---|---|---|---|---|---|
| Phylum | Bacteroidetes | ↑ | + | − | + | ns |
| Family | Bifidobacteriaceae | ↑ | + | − | + | ns |
| Family | Lachnospiraceae | ↓ | − | + | − | ns |
| Genus | *Bifidobacterium* | ↑ | + | − | + | ns |

**Footnote:** Results are exploratory; no taxa remained significant after multiple-testing correction.
